# Supplementary material for: Mode selection mechanism in traveling and standing waves revealed by Min wave reconstituted in artificial cells
Source: Sci Adv. 2022 Jun 8;8(23):eabm8460. doi: 10.1126/sciadv.abm8460 (PMC9177070; doi:10.1126/sciadv.abm8460)
Supplement: Supplementary file 1 — Figs. S1 to S10 [file sciadv.abm8460_sm.pdf]

Science Advances
AAAS

Supplementary Materials for

Mode selection mechanism in traveling and standing waves revealed by Min wave reconstituted in artificial cells

Sakura Takada *et al.*

Corresponding author: Natsuhiko Yoshinaga, yoshinaga@tohoku.ac.jp; Kei Fujiwara, fujiwara@bio.keio.ac.jp

Sci. Adv. **8**, eabm8460 (2022)
DOI: 10.1126/sciadv.abm8460

The PDF file includes:

Figs. S1 to S10
Legends for movies S1 to S10

Other Supplementary Material for this manuscript includes the following:

Movies S1 to S10

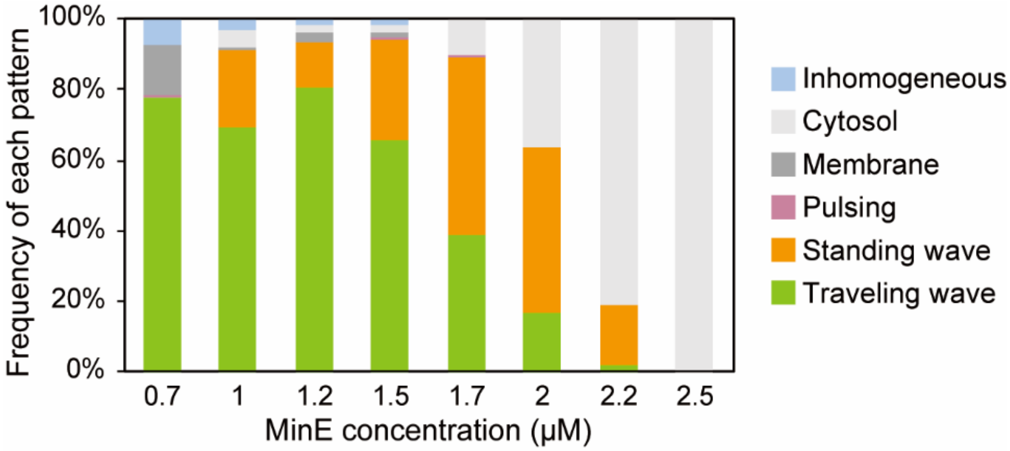

Fig. S1. Frequencies of each pattern generated by the Min proteins in artificial cells at various MinE concentrations

MinE concentrations are varied from 0.7 to 2.5 μM, and MinD concentration is fixed at 1 μM (n = 160-217). The details of each pattern are described in the main text.

	concentration (msfGFP and mCherry)			
	0.4 μM	1 μM	2 μM	4 μM
ratio (Ave ± SD)	0.934 ± 0.39	1.047 ± 0.046	0.999 ± 0.036	1.000 ± 0.039
ratio (SD/Ave)	4.13%	4.37%	3.65%	3.88%
n	106	263	306	373

Fig. S2. Variation in protein numbers among different artificial cells

A solution in which msfGFP and mCherry are mixed in equimolar (0.4 – 4.0 μM each) were encapsulated in artificial cells by the same procedure as Min wave assays, and fluorescent signals in each artificial cells were captured by using a confocal microscope (FV1000, Olympus, Tokyo, Japan) after encapsulation. The ratio of these proteins was semi-automatically quantified by using Analyze Particle method of Fiji software. Averages and standard deviations of the concentration ratio of msfGFP to mCherry in artificial cells are shown. Concentrations were determined by calibration curves obtained by the average intensities of 0.4, 1, 2, 4 μM msfGFP or mCherry.

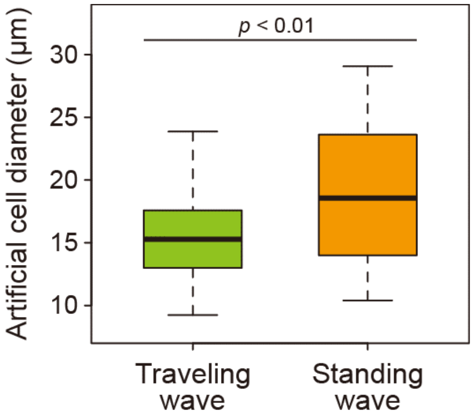

Fig. S3. Size dependence of the Min wave modes

The relationship between each wave mode and diameters of artificial cells under 1 μM MinD and MinE condition is plotted on the boxplot (traveling wave: n = 84, standing wave: n = 24). The p-value describes the result of unequal variances t-test.

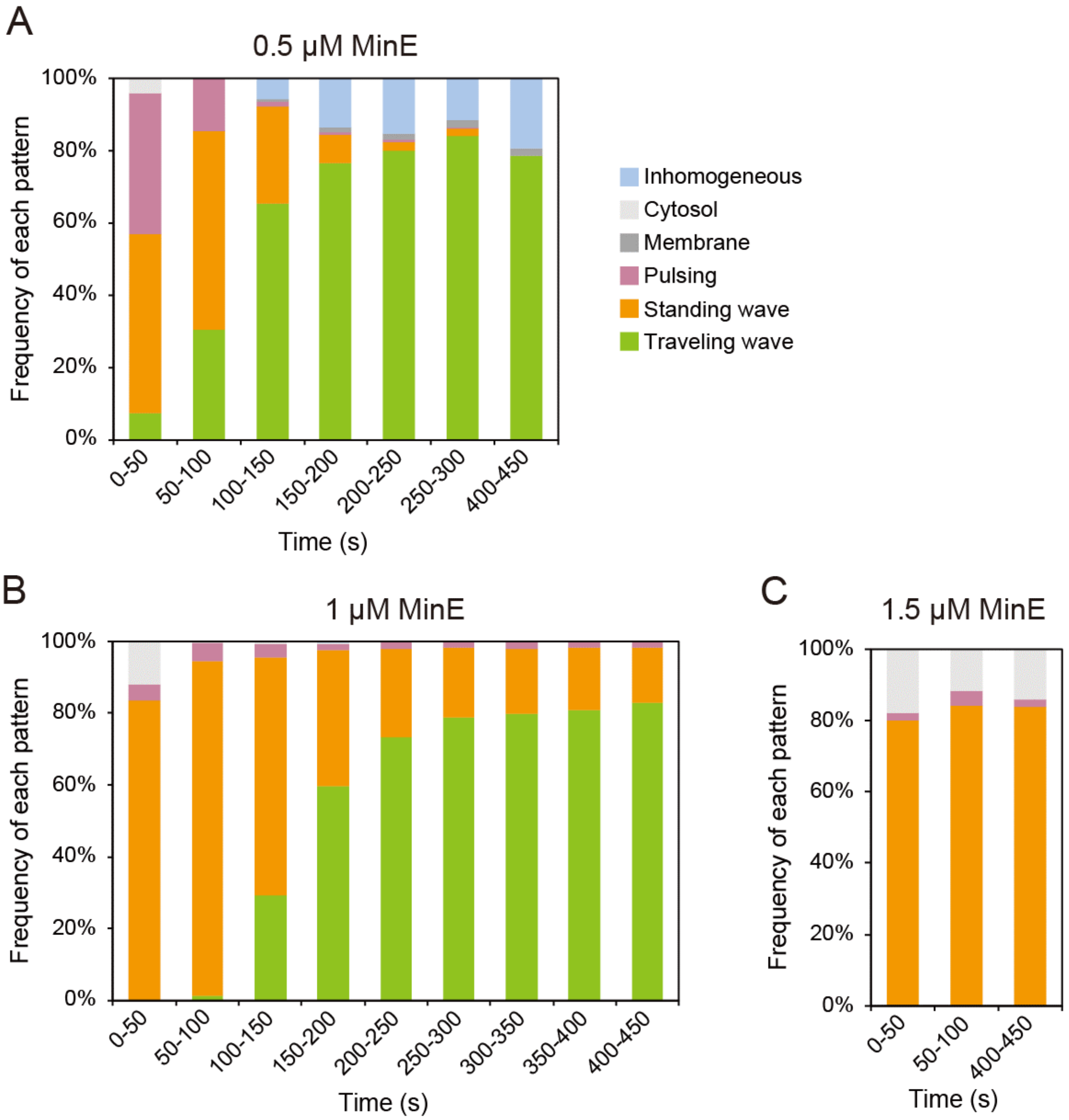

Fig. S4. Time development of the frequencies of each pattern generated by the Min system in artificial cells

MinE concentrations are (**A**) 0.5 (n = 150-165), (**B**) 1.0 (n = 218-332), and (**C**) 1.5 μM (n = 90-99).

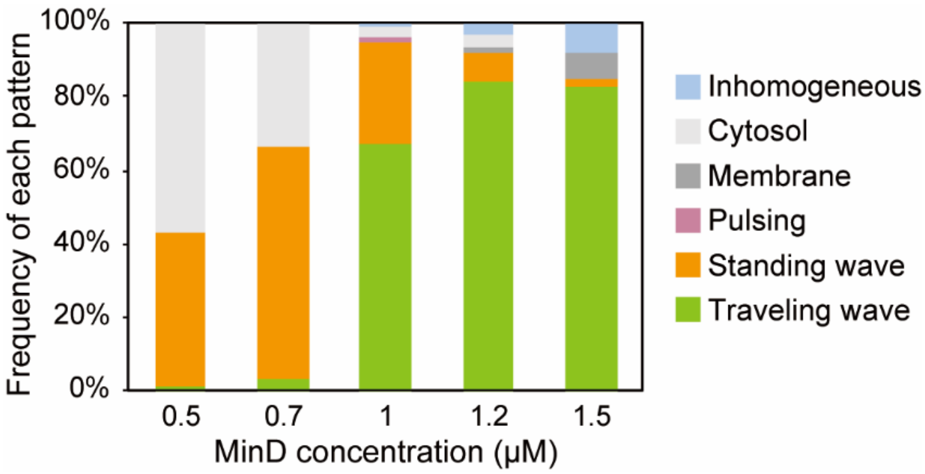

Fig. S5. Frequencies of each pattern generated by the Min proteins in artificial cells at various MinD concentrations

MinD concentrations are varied from 0.5 to 1.5 μM, and MinE concentration is fixed at 1 μM (n = 158-249).

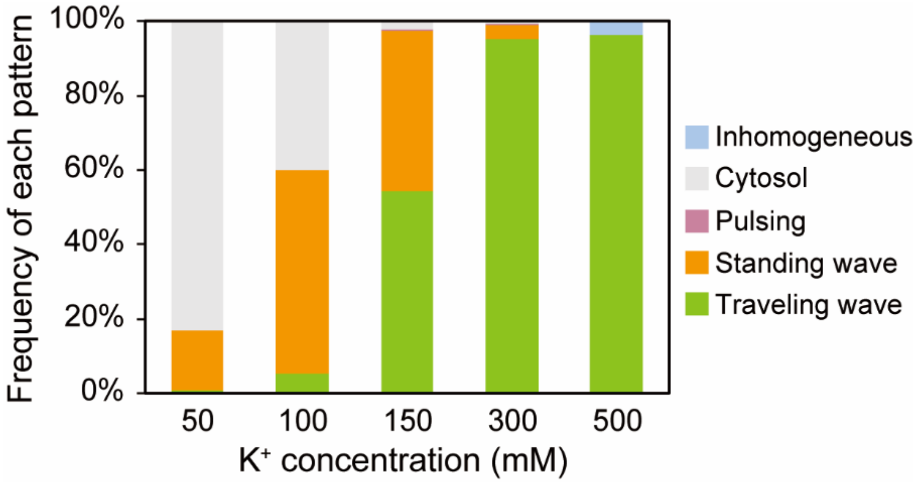

Fig. S6. Frequencies of each pattern generated by the Min system in artificial cells at various K^+ concentration

Concentrations of MinD and MinE are 1 μM (n = 130-213). K^+ concentration indicates GluK concentration in the reaction buffer (See Materials and Methods).

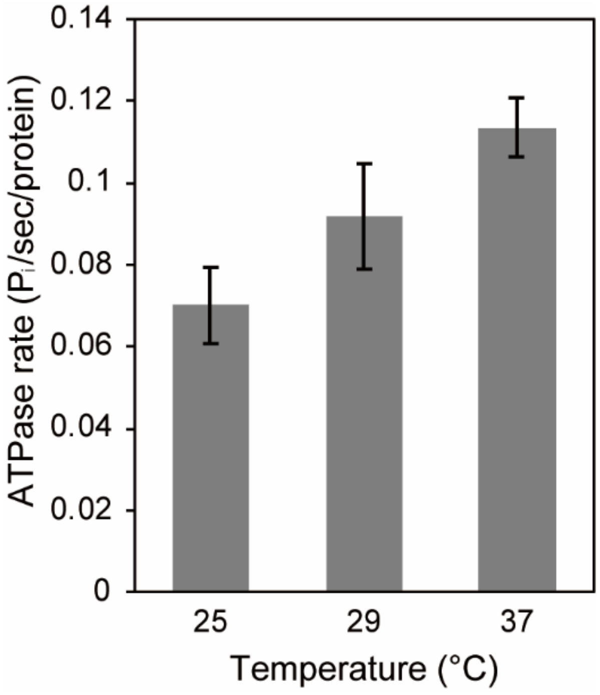

Fig. S7. ATPase rates of MinDE complex with increasing temperature
Mean ± standard error (n = 4) are shown.

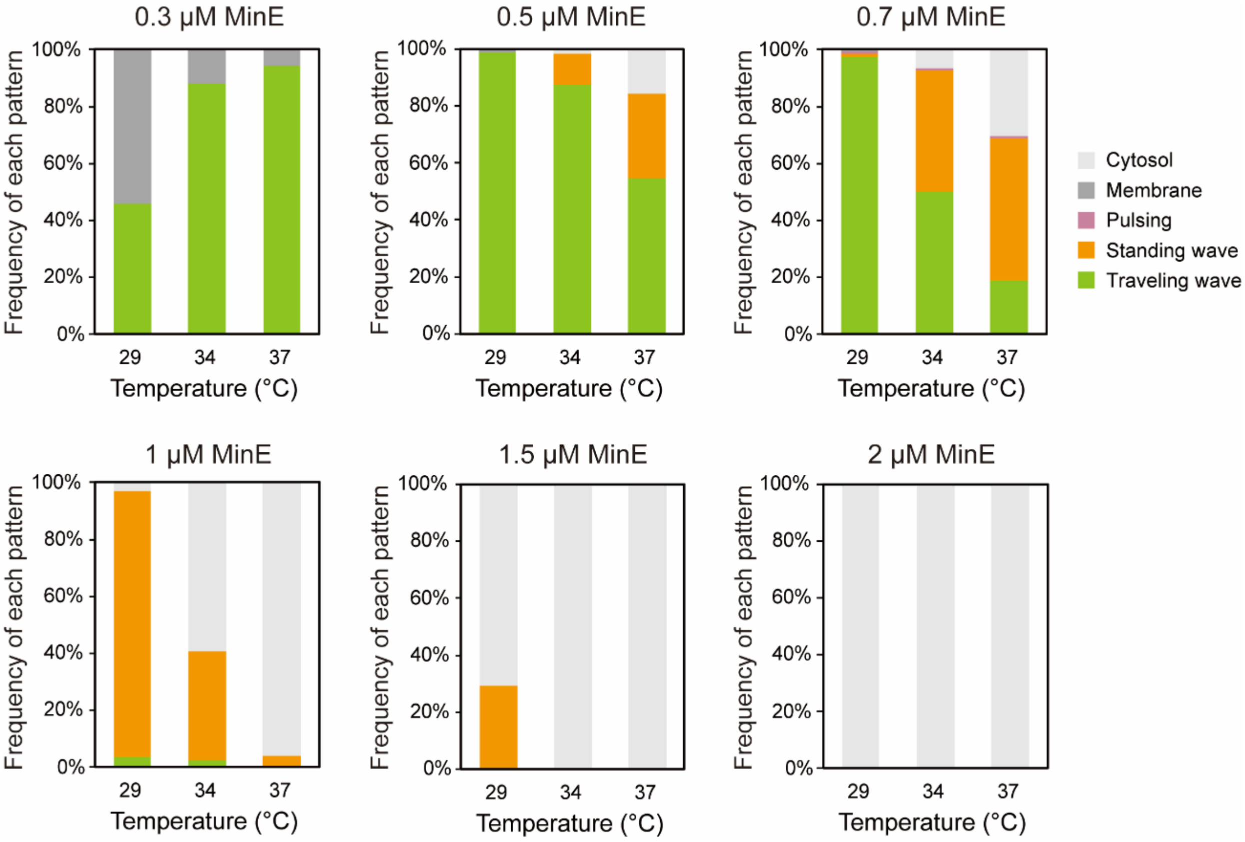

Fig. S8. Temperature dependence of frequencies of each pattern generated by the Min proteins in artificial cells at various MinE concentrations

Temperatures are at 29, 34, 37°C, and MinE concentrations are from 0.3 to 2 μM (n = 88-162, 0.7 μM MinE at 37°C: n = 280).

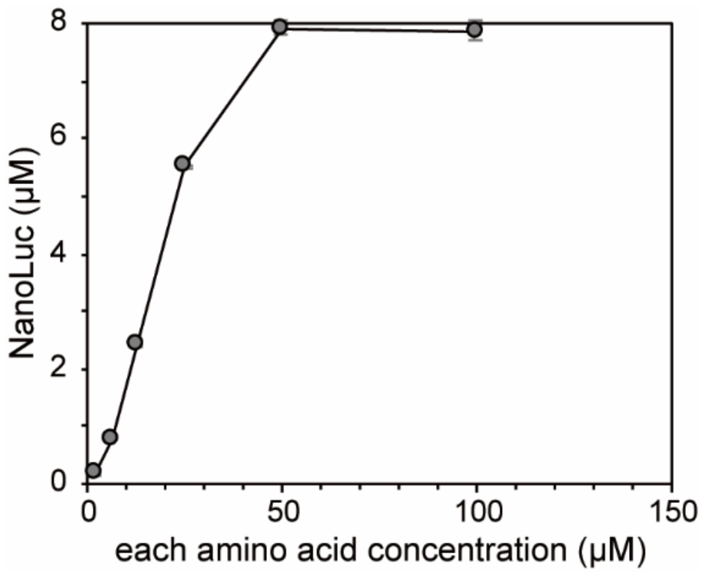

Fig. S9. Expression levels of NanoLuc by PURE system at various amino acid concentrations

The amount of NanoLuc synthesized by PURE system depends on each amino acid concentration. Mean ± standard deviation (n = 3) are shown.

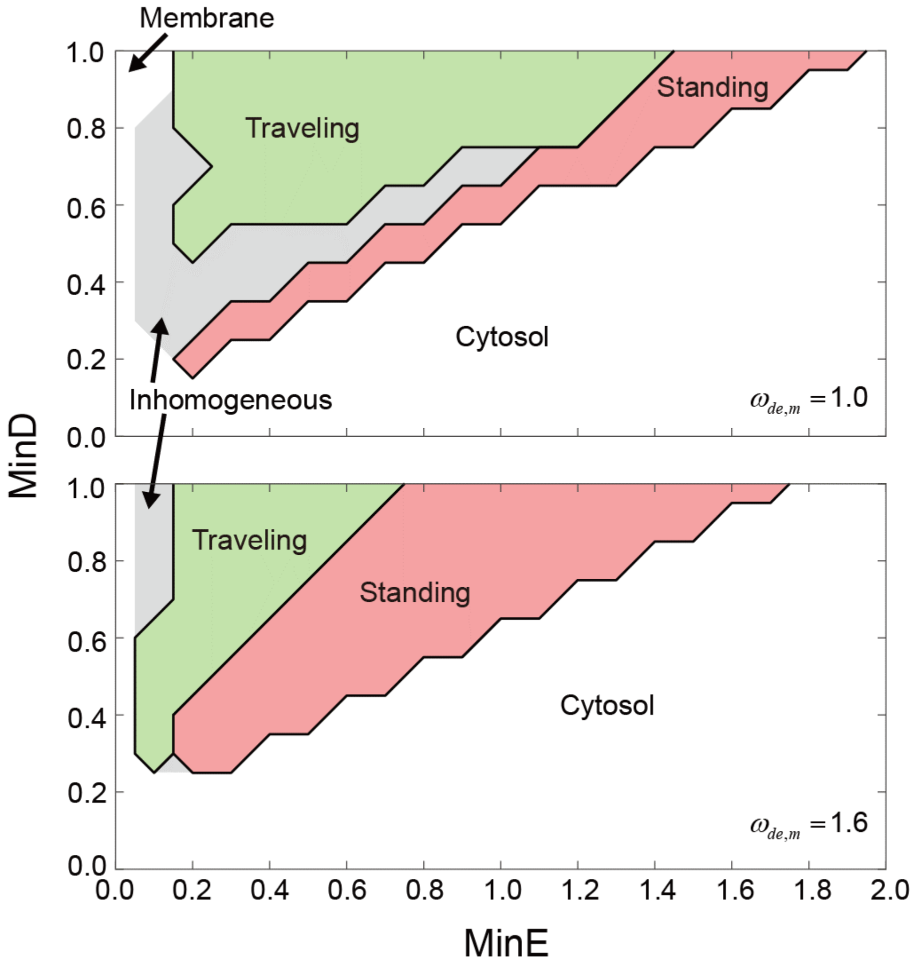

Fig. S10. Selection of standing and traveling waves in the three-dimensional theoretical model

(**A**, **B**) State diagrams of the generated waves under varying MinE-MinD concentrations at (**A**) and (**B**). The MinD and MinE concentrations are non-dimensionalized numbers in simulations (the same as those in Fig 5).

Movie S1.
Min wave in artificial cells with different MinE concentrations (scale bar: 10 μm)

Movie S2.
Initial Min wave mode transition at 0.5 μM MinE

Movie S3.
Initial Min wave mode transition at 1.0 μM MinE

Movie S4.
Initial Min wave mode transition at 1.5 μM MinE

Movie S5.
Min wave in artificial cells with different MinD concentrations (scale bar: 10 μm)

Movie S6.
Min wave in artificial cells with different potassium concentrations (scale bar: 10 μm)

Movie S7.
Min wave mode transition by temperature shift (scale bar: 10 μm)

Movie S8.
Min wave mode transitions by temperature shifts (scale bar: 10 μm)

Movie S9.
Min waves in artificial cells with MinE synthesis (1.5 μM aa)

Movie S10
Min waves in artificial cells with MinE synthesis (2 μM aa)
